# Emotional stabilization interventions for people exposed to chronic traumatic events, in humanitarian settings

**DOI:** 10.1192/j.eurpsy.2023.429

**Published:** 2023-07-19

**Authors:** E. Dozio

**Affiliations:** Action contre la Faim, Paris, France

## Abstract

**Introduction:**

In contexts of chronic crisis, such as wars, the population is affected by prolonged traumatic exposure. In this type of context, it is sometimes difficult to provide psychological support to work through the trauma because the environment is neither stable nor reassuring. It is about finding care devices that despite the complexity of the situation, manage to bring relief and improve the ability to manage negative emotions and stress.

**Objectives:**

The objective of the intervention was to relieve people in distress, to help them contain their reactions to moments of anxiety so that they are more available and calm in their daily lives. Since the intervention could not provide long-term treatment for the potential traumatic stress, due to the abuses still in progress, it aimed to prevent the appearance and installation of the symptoms.

**Methods:**

A group protocol based on emotional stabilization exercises was offered to children and adults from communities affected by the fighting in the civil war in the Central African Republic in 2021. In addition to giving elements of psychoeducation, participants could practice exercises aimed at stabilizing emotions and managing stress.

People could benefit from this psychosocial support with daily frequency for four consecutive days. At the beginning and at the end of the care device, psychometric scales were administered in order to be able to measure the improvement in well-being (WHO5) the reduction in symptoms of anxiety and depression (HAD) as well as traumatic symptoms in adults (PCL -5) and children (CPTS-RI).

**Results:**

Between February and April 2021, 1,200 adults and 400 children were able to participate in the emotional stabilization device. 90% of the participants showed an improvement in well-being and a reduction in stress and axiety reactions. The participants particularly appreciated the exercises for the ease with which they could be reproduced in daily life and transmitted to other members of the family and the community. Despite the fact that exposure to stress remained significant and daily, they expressed the feeling of having regained some control, at least over their own emotions and reactions.

**Conclusions:**

In situations of chronic stress, where psychic traumatic symptoms cannot be treated sustainably, this emotional stabilization protocol can be an effective option to regulate the emotional states of exposed people and give them tools to cope with anxiety.

Two important points to emphasize are also the fact that this device can reach a large part of the population without a lot of means and that it can be provided by non-professionals in mental health, if properly trained and constantly supervised by experts.

**Disclosure of Interest:**

None Declared

